# The Efficacy of Contrast Transthoracic Echocardiography and Contrast Transcranial Doppler for the Detection of Patent Foramen Ovale Related to Cryptogenic Stroke

**DOI:** 10.1155/2020/1513409

**Published:** 2020-05-27

**Authors:** Jing Yang, Huiqin Zhang, Yumeng Wang, Shiquan Zhang, Tingyu Lan, Meng Zhang, Yuanzi Li, Wenyan Huang, Hongxia Zhang, Anxin Wang, Yang Xiao, Lijuan Du

**Affiliations:** ^1^Department of Ultrasound, Beijing Tiantan Hospital, Capital Medical University, 119 West Road of South 4th Ring Road, Fengtai District, Beijing, China 100160; ^2^Shenzhen Institute of Advanced Technology, Chinese Academy of Sciences, 1068 Xueyuan Avenue, Shenzhen University Town, Nanshan District, Shenzhen, China 518055; ^3^China National Clinical Research Center for Neurological Diseases, Beijing Tiantan Hospital, Capital Medical University, 119 West Road of South 4th Ring Road, Fengtai District, Beijing, China 100160

## Abstract

**Background:**

Patent foramen ovale (PFO) has been linked to the pathophysiology of cryptogenic stroke. Contrast transesophageal echocardiography (cTEE) is the current gold standard for PFO diagnosis, but it has the disadvantage of being semi-invasive and does not exempt from risks. As a diagnostic test, the efficacy of contrast transthoracic echocardiography (cTTE) and contrast transcranial Doppler (cTCD) is controversial. This study is aimed at investigating the efficacy of cTTE and cTCD versus cTEE in PFO detection, exploring a more cost-effective and reliable method for the diagnosis of PFO related to cryptogenic stroke.

**Methods:**

From August 2019 to January 2020, a total of 213 patients with suspected PFO were included in our study. All patients underwent cTEE, cTCD, and cTTE examinations. cTTE3 was named for using a cutoff of 3 beats to detect PFO during cTTE, and cTTE5 represented a cutoff of 5 beats. A cutoff of cTCD grade III was named cTCD III. A cutoff of grade IV was named cTCD IV. cTTE3+cTCD IV was used for the combination of a cutoff of 3 beats during cTTE with grade IV of cTCD. cTTE5+cTCD III combined a cutoff of 5 beats during cTTE with cTCD grade III. Taking cTEE as the gold standard, we compared the sensitivity, specificity, negative likelihood ratio (-LR), and misdiagnosis rate for PFO detection among the above methods.

**Results:**

A total of 161 of 213 (76%) patients had PFO confirmed by cTEE. With the spontaneous Valsalva maneuver, the sensitivity, specificity, negative likelihood ratio (-LR), and misdiagnosis rate of cTTE3 in PFO diagnosis were 60%, 90%, 44%, and 10%, respectively, and those for cTTE5 were 76%, 78%, 31% and 22%, respectively. The sensitivity, specificity, negative likelihood ratio (-LR), and misdiagnosis rate of cTCD III were 80%, 71%, 29%, and 29%, respectively, while those for cTCD IV were 55%, 90%, 49%, and 10%, respectively. When cTTE and cTCD were combined to diagnose PFO, the specificity and misdiagnosis rate were significantly improved, especially cTTE3+cTCD IV, with 100% specificity and a misdiagnosis rate of 0.

**Conclusion:**

cTTE or cTCD can be used for preliminary PFO related to cryptogenic stroke findings. The combination of the two methods can improve the specificity of PFO diagnosis, especially using the cutoff of cTTE3+cTCD IV.

## 1. Introduction

The foramen ovale is a form of interatrial communication in the fetal circulation. At birth, with the development of the lungs, which increases the pressure gradient from left to right, the foramen ovale is functionally closed. PFO is defined as the foramen ovale not fusing when children are older than 3 years [1–3]. The overall incidence is between one-quarter and one-third of individuals [4, 5]. Recent studies have confirmed that PFO is a risk factor for cerebral events, such as cryptogenic stroke, transient ischemic attacks (TIA), migraine headaches, and hypoxemia [6–8]. PFO may act as the pathway of thrombi from the venous circulation to the cerebral circulation and could cause paradoxical embolisms, even inducing transient arrhythmia, both of which are recognized as potential underlying mechanisms [7, 9, 10]. CTEE has become the main means of PFO detection, but it has the disadvantage of being semi-invasive and dose not exempt from risks. As noninvasive methods, cTTE and cTCD are used as screening tests for PFO, but the reliability for PFO diagnosis is controversial [11–13]. This study is aimed at investigating the efficacy of cTTE and cTCD versus cTEE to detect PFO, exploring a more cost-effective and reliable method for the diagnosis of PFO related to cryptogenic stroke.

## 2. Materials and Methods

### 2.1. Study Population and Clinical Assessment

A total of 213 patients suffering from migraines, stroke, or vertigo disease and suspected PFO at Beijing Tiantan Hospital Affiliated with Capital Medical University between August 2019 and January 2020 were involved. The inclusion criteria were as follows: (1) aged 18-65 years; (2) volunteered to participate in the study; (3) all underwent cTEE, cTTE, and cTCD; and (4) good image quality during all the examinations. The exclusion criteria were as follows: (1) congenital heart disease (CHD), such as atrial septal defect (ASD) and ventricular septal defect (VSD); (2) patients who could not perform the Valsalva maneuver; (3) images were not clear during cTTE or cTCD examinations; and (4) refused to participate in the study. The study was approved by the ethics committee of the hospital institution. All enrolled participants signed an informed written consent form.

### 2.2. Contrast Transesophageal Echocardiography (cTEE)

**Figure 1:**
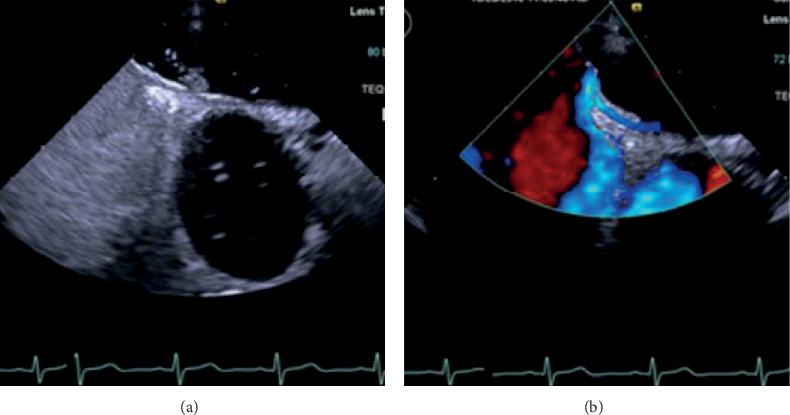
Contrast transesophageal echocardiographic (cTEE) images of PFO. PFO was detected with right-to-left shunting (a) or flow signal (b).

Before the examinations, all patients were thoroughly instructed and trained to perform the Valsalva maneuver. All patients received local pharyngeal anesthesia for 10 minutes (dyclonine hydrochloride mucilage 10 ml). The examinations were performed by the same experienced doctors. Standard echocardiographic protocols were followed on the basis of the recommendations of the American Society of Echocardiography [14]. The saline contrast was produced by 1 ml of air, 1 ml of blood, and 8 ml of saline. The content was agitated between two 10 ml syringes connected with a three-way stopcock (≥20 times) and was injected rapidly from the right antecubital vein approximately 5 seconds after starting the Valsalva maneuver [15]. The presence of PFO was confirmed when microbubbles or color Doppler flow signal crossed from the foramen ovale (Figure 1).

### 2.3. Contrast Transthoracic Echocardiography (cTTE)

**Figure 2:**
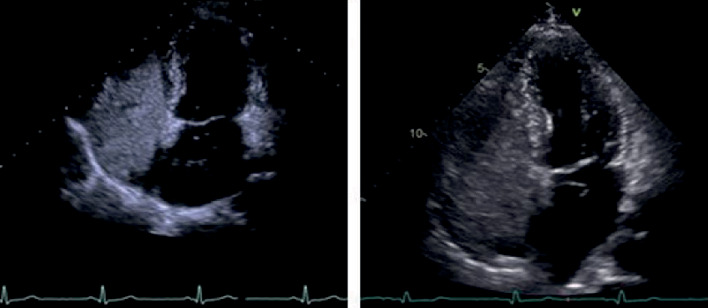
Contrast transthoracic echocardiographic (cTTE) images of PFO, clearly showing the interatrial septum and the agitated saline contrast.

cTTE examinations were carried out by experienced sonographers using either 3-5 MHz multiplane transducers produced by GE or Siemens. Standard echocardiographic protocols were followed on the basis of the recommendations of the American Society of Echocardiography [16]. An apical four-chamber view was acquired, and the gain settings were individually adjusted to optimize the visualization of the interatrial septum and the agitated saline contrast (Figure 2). The agitated saline was then promptly injected into the patients' veins, and the operation was consistent with cTEE. The cardiac cycles of microbubbles that appeared in the left atrium were calculated from full right atrial opacification with agitated saline.

### 2.4. Contrast Transcranial Doppler (cTCD)

cTCD examinations were completed by professional departments and performed according to a uniform guideline [17]. The shunt was determined to be grade 0 (no microembolic signal), grade I (1–10 microembolic signals), grade II (11-30 microembolic signals), grade III (31-100 microembolic signals), or grade IV (>100 or with “curtain”) [17].

### 2.5. Statistical Analysis

Statistical analysis was performed using commercially available statistical software (SPSS version 22.0). Continuous variables were expressed as the mean ± SD and percentages. Comparisons for variables were analyzed using *t*-tests, *χ*^2^-tests, and rank sum tests. *P* values < 0.05 were considered statistically significant.

## 3. Results

### 3.1. Patients' Characteristics

The general characteristics of the patients are shown in Table 1. A total of 161 patients had PFO confirmed by cTEE. Subjects were divided into two groups: PFO(+) and PFO(-). There were no significant differences in age or etiology between the two groups (*P* > 0.05), but there were more female patients in the PFO(+) group (*P* < 0.05).

### 3.2. Contrast Transthoracic Echocardiography for PFO Detection

With the spontaneous Valsalva maneuver, microbubbles appeared in the left atrium in 3 cardiac cycles after full right atrial opacification for the cTTE3 group and 5 cardiac cycles for the cTTE5 group. The sensitivity, specificity, negative likelihood ratio (-LR), and misdiagnosis rate of the two groups are shown in Table 2. When 3 cardiac cycles were used as the cutoff value for a positive result, the sensitivity and specificity for detecting PFO were 60% and 90%, respectively, but for 5 cardiac cycles, the sensitivity and specificity were 76% and 78%, respectively. The -LR and misdiagnosis rates of cTTE3 were 44% and 10%, respectively, and those of cTTE5 were 31% and 22%, respectively.

### 3.3. Contrast Transcranial Doppler for PFO Detection

The classifications of the cTCD test in 213 subjects are shown in Table 3. When grade cTCD ≥ III (>30 microbubbles) was used as the cutoff value, the sensitivity for detecting PFO was 80%, the specificity was 71%, the negative likelihood ratio (-LR) was 29%, and the misdiagnosis rate was 29%. When the grade was ≥IV (>100 microbubbles or with “curtain”), and cTCD IV was used as the cutoff value, the sensitivity for detecting PFO was 55%, the specificity was 90%, the negative likelihood ratio (-LR) was 49%, and the misdiagnosis rate was 10%. The results are shown in Table 4.

### 3.4. PFO Detection by Combining the Diagnosis with CTTE and CTCD

When the cutoff value of PFO detection was 3 cardiac cycles during cTTE combined with cTCD grade IV (cTTE3+cTCD IV), the sensitivity was 39%, the specificity was 100%, the -LR was 61%, and the misdiagnosis rate was 0. The cutoff value for 5 cardiac cycles during cTTE combined with cTCD grade III (cTTE5+cTCD III) had a sensitivity of 63%, a specificity of 94%, a -LR of 39%, and a misdiagnosis rate of 6%. The results are shown in Table 5. We found that the specificity was significantly improved when cTTE was combined with cTCD for detecting PFO. The cTTE3+cTCD IV had the highest specificity and -LR, meanwhile the lowest misdiagnosis rate. However, cTTE5+cTCD III had relatively higher accuracy.

## 4. Discussion

The major findings of our present study were as follows: (1) ≥grade III cTCD was a strong evidence for a positive result for PFO detection, associated with cryptogenic stroke. (2) The specificity was significantly improved if the two methods (cTTE and cTCD) were combined for PFO detection, but their combined use was associated with a drop in sensitivity. (3) cTTE5+cTCD III was more accurate and stable than cTTE3+cTCD IV, while cTTE3+cTCD IV had greater specificity and misdiagnosis rate.

Stroke has brought serious consequences and an economic burden for families. Individuals with migraine are at a higher risk for stroke [18]. Recent studies have demonstrated that PFO is implicated in stroke, especially cryptogenic stroke, and the prevalence of PFO in patients with cryptogenic stroke is much higher than that in the normal healthy population [7]. Transcatheter closure of PFO is superior to medical therapy in reducing the recurrence of stroke [19–21]. The issue of PFO detection has become the focus of increasing interest. Currently, cTEE plays the main role in the evaluation of PFO. But application of cTEE is limited. It has the disadvantages of being uncomfortable and some risks. Although unusual, severe complications such as esophageal bleeding or perforation may occur. In addition to the contraindications for cTEE, such as esophageal varices, Barrett's esophagus, pharyngeal carcinoma, or patients with a serious bleeding risk, it is important to have a reliable alternative in contemporary clinical practice.

In contrast, cTTE and cTCD have the advantage of being noninvasive and having a lower cost. Although the methods have been used in many hospitals, the results of tests vary considerably [11–13]. Some studies have found that microbubbles appearing in the left chambers in 3 or 5 cardiac cycles after full right atrial opacification during cTTE were related to PFO [16, 22, 23]. In our study, the 5 cardiac cycles during cTTE had better accuracy and stability, a sensitivity of 76%, and a specificity of 78%. The appearance of bubbles in the left atrium may be influenced by the length of the oval valve and the pressure difference between the two atriums after the Valsalva maneuver, so the timing of microbubbles passing through the PFO is different, possibly longer cardiac cycles can obtain more information. cTCD also provides good patient tolerance and excellent accuracy, and it is a useful alternative for detecting PFO, especially grade ≥ III in our study. Comparing cTTE with cTCD in detecting PFO (cTTE3 vs. cTCD IV, cTTE5 vs. cTCD III), the sensitivity and specificity were not statistically significant. This result indicated that the function of cTEE and cTCD may be equal in PFO detection. We speculated that the reason may be related to the division of threshold values in our study. Therefore, we combined them further. Finally, we found that when the two methods were combined, the specificity and misdiagnosis rate were significantly improved, especially the cutoff value of 3 cardiac cycles during cTTE combined with cTCD grade IV (cTTE3+cTCD IV), with a specificity of 100% and a misdiagnosis rate of 0. Specificity is the percentage of subjects diagnosed with negative results in the diagnostic test, also known as true negative, which indicated that joint inspection had the key filtering function for patients with suspected in diagnosing PFO.

However, in terms of sensitivity alone, single inspection was more significant. A higher sensitivity means a lower misdiagnosis rate, which is helpful for excluding corresponding diseases. Therefore, for PFO detection, we must comprehensively consider the choice regarding the above methods. If stroke is highly suspected, emphasis is placed on specificity, but if only clinical screening, then on sensitivity.

Additionally, in our study, we found a difference between the sexes in the PFO(+) and PFO(-) groups. Because there were 150 migraineurs, accounting for 70% of all participants, 68% of which were female, the sex difference may be related to a lower pain threshold in women [24, 25].

## 5. Study Limitations

This study defined the appearance of microbubbles within 3 or 5 cardiac cycles as a positive result on cTTE, but microbubbles may appear after five cardiac cycles in very few patients with PFO.

## 6. Conclusion

In conclusion, cTTE and cTCD play an important role in detecting PFO related to cryptogenic stroke. Although the sensitivity with combined inspection is not high enough, it has a high specificity and is still possible to guide more rational application of cTEE in diagnosing PFO, related to cryptogenic stroke.

## Figures and Tables

**Table 1:** Patient characteristics (*N* = 213).

Characteristics	PFO(+) (*n* = 161)	PFO(-) (*n* = 52)
Age (y)	41 ± 12	44 ± 14
Female	103	42
Cryptogenic stroke	33	16
Migraine	116	34
Vertigo disease	12	2

Age and etiological distribution were not statistically significant in the two groups, but there were more women in the PFO(+) group.

**Table 2:** The efficacy of cTTE for PFO detection.

	Sensitivity (%)	Specificity (%)	-LR (%)	Misdiagnosis rate (%)
cTTE3	60	90	44	10
cTTE5	76	78	31	22

Compared the efficacy of 3 with 5 cardiac cycles as the cutoff value in the cTTE test. Each had its advantages, but the accuracy of cTTE5 was better.

**Table 3:** The classifications of the cTCD test in the two groups.

	Grade 0	Grade I	Grade II	Grade III	Grade IV	Total
PFO(+)	1	14	18	39	89	161
PFO(-)	3	22	13	10	4	52
Total	4	36	31	49	93	213

The classification distribution of the two groups was statistically significant, especially grades III and IV.

**Table 4:** The efficacy of cTCD for PFO detection.

	Sensitivity (%)	Specificity (%)	-LR (%)	Misdiagnosis rate (%)
cTCD III	80	71	29	29
cTCD IV	55	90	49	10

Compared the efficacy of grade III with IV as the cutoff value in the cTCD test. Each had its advantages, but the accuracy of cTCD III was better.

**Table 5:** The efficacy of cTTE+cTCD for PFO detection.

	Sensitivity (%)	Specificity (%)	-LR (%)	Misdiagnosis rate (%)
cTTE5+cTCD III	63	94	39	6
cTTE3+cTCD IV	39	100	61	0

Compared the efficacy of 5 cardiac cycles and grade III with 3 cardiac cycles and grade IV as the cutoff. The specificity was improved significantly when the two methods (cTTE and cTCD) were combined for PFO detection. The accuracy of cTTE5+cTCD III was better.

## Data Availability

The materials in this manuscript are available from the corresponding author on reasonable request.
